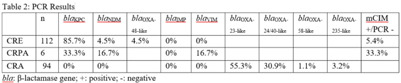# Characterization of carbapenem-resistant gram-negative bacteria collected in the Sentinel Surveillance Program, 2018–2019

**DOI:** 10.1017/ash.2022.156

**Published:** 2022-05-16

**Authors:** Lori Spicer, Davina Campbell, J. Kristie Johnson, Cynthia Longo, Thomas Balbuena, Thomas Ewing, Maria Karlsson, J. Kamile Rasheed, Christopher Elkins, Amy Gargis, Joseph Lutgring

## Abstract

**Background:** Carbapenem resistance in gram-negative organisms is an important public health problem. The CDC conducted Sentinel surveillance in 2018–2019 to characterize these organisms from 9 facilities in 9 different states. **Methods:** Carbapenem-resistant Enterobacterales (CRE), *Pseudomonas aeruginosa* (CRPA), and *Acinetobacter* spp (CRA) obtained from clinical samples of patients in acute-care or long-term care facilities were submitted to the CDC. Identification was confirmed using matrix-assisted laser desorption ionization time-of-flight (MALDI-TOF), and antimicrobial susceptibility testing (AST) was performed via broth microdilution for 27 antibiotics. All confirmed CRE and CRPA were tested for carbapenemase production (CP) using the modified carbapenem inactivation method (mCIM). The isolates that were mCIM-positive were assessed by real-time PCR for presence of *bla*KPC, *blaN*DM, *bla*VIM, and *bla*IMP. CP-CRE were also assessed for *bla*OXA-48-like. All confirmed CRA were tested for the same genes as CRPA and *bla*OXA-23–like, *bla*OXA-24/40-like, *bla*OXA-58–like, and *bla*OXA-235–like genes. Difficult-to-treat resistance (DTR) was defined as resistance to all β-lactams (excluding newer β-lactam combination agents) and quinolones tested. **Results:** The CDC confirmed 208 CRE, 161 CRPA, and 94 CRA. Table 1 summarizes AST results for a selection of drugs. We identified 112 (53.8%) mCIM-positive CRE and 6 (3.7%) mCIM-positive CRPA. The PCR results are summarized in Table 2. One mCIM-positive and PCR-negative isolate was positive in a metallo-β-lactamase screen. **Conclusions:** Resistance among CRE and CRPA to newer β-lactam combination agents was detected. Options for treating CRA are limited. Of 112 CP-CRE, 85.7% harbored *bla*KPC; CP-CRPA were rare (3.7%); and most CRA harbored *bla*OXA-23-like (55.3%) or *bla*OXA-24/40-like (30.9%). Whole-genome sequencing is planned to better understand gene variants, sequence types, and additional resistance markers present among the isolates.

**Funding:** None

**Disclosures:** None

**Table:**
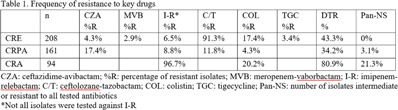

**Table:**